# Ostrowski type inequalities for sets and functions of bounded variation

**DOI:** 10.1186/s13660-017-1429-5

**Published:** 2017-06-26

**Authors:** Oleg V Kovalenko

**Affiliations:** Department of Mechanics and Mathematics, O. Honchar Dnipro National University, pr. Gagarina, 72, Dnipro, 49010 Ukraine

**Keywords:** 26D15, 41A17, 41A44, 41A63, Ostrowski type inequalities, multivariate functions of bounded variation, sets of bounded variation

## Abstract

In this paper we obtain sharp Ostrowski type inequalities for multidimensional sets of bounded variation and multivariate functions of bounded variation.

## Introduction

In 1937 Ostrowski [1] proved the following inequality.

### Theorem

*Let* $f\colon[0,1]\to \mathbb {R}$ *be a differentiable on* $(0,1)$ *function with bounded on* $(0,1)$ *derivative*. *Then*, *for all* $x\in[0,1]$,

$$\biggl\vert \int _{0}^{1}f(t)\,dt - f(x)\biggr\vert \leq \biggl( \frac {1}{4} + \biggl( x-\frac{1}{2} \biggr) ^{2} \biggr) \sup_{t\in(0,1)}\bigl\vert f'(t)\bigr\vert . $$

*The inequality is sharp*.

Inequalities that estimate deviation of a function from its mean value using different characteristics of the function are usually called Ostrowski type inequalities. Such inequalities have many applications, in particular in the area of numerical methods, and are heavily studied. See [2] and the references therein for results connected with Ostrowski type inequalities for univariate functions of bounded variation and their applications.

The goal of this article is to obtain sharp Ostrowski type inequalities for multivariate functions and multidimensional sets of bounded variations. There are several ways to extend the notion of bounded variation to multivariate functions, see [3] for a review of different approaches for functions of two variables; [4] for the point of view that is generally accepted in literature now.

We introduce a new definition of bounded variation that is based on the Kronrod-Vitushkin approach [5]. The introduced variation of a multivariate function has (unlike any of the Kronrod-Vitushkin variations) the following two properties: the variation does not change if the argument of the function is multiplied by a non-zero constant; and the variation of a multivariate radial function is twice bigger than the variation of the generating one-dimensional function, see Properties 9 and 12 below for rigorous statements of the properties.

The paper is organized as follows. In Section 2 we list the notations used throughout the paper. In Sections 3 and 4 we introduce definitions, justify the correctness of the definitions, and list some properties of the sets and function variations. Section 5 is devoted to Ostrowski type inequalities.

## Notations

For a set $F\subset\mathbb{R}^{d}$, denote by *∂F*, int*F* and *F̅* its boundary, interior and closure, respectively. For arbitrary $t\in \mathbb {R}$, set $\mathbb{R}^{d}_{t}:=\{(x,t)\in\mathbb {R}^{d}\colon x\in \mathbb {R}^{d-1}\}$. For $x,y\in\mathbb{R}^{d}$, by *xy* we denote the segment with ends in the points *x* and *y*, i.e., $xy=\{(1-t)x+ty\colon t\in[0,1]\}$. For $c\in \mathbb {R}$ and $F\subset\mathbb{R}^{d}$, set $cF:=\{cx\colon x\in F\}$.

For two sets $F_{1},F_{2}\subset\mathbb{R}^{d}$, set $\rho(F_{1},F_{2}):= \inf_{x\in F_{1}, y\in F_{2}}\vert x-y\vert $, where $\vert w\vert $ denotes the Euclidean distance between the point $w\in\mathbb{R}^{d}$ and zero element *θ* of $\mathbb{R}^{d}$.

For $\varepsilon\geq0$, two sets $F_{1},F_{2}\subset\mathbb{R}^{d}$ are called *ε*-disjoint if $\rho(F_{1}, F_{2})>\varepsilon$. Obviously, two compact sets $F_{1}, F_{2}\subset\mathbb{R}^{d}$ are 0-disjoint if and only if they are disjoint. For $\varepsilon>0$, a set *F* is called *ε*-connected if there does not exist a partition $F= F_{1}\cup F_{2}$ into *ε*-disjoint non-empty sets $F_{1}$, $F_{2}$. Some properties of *ε*-components can be found in Appendix I of [6].

For an arbitrary function $f\colon E\subset\mathbb{R}^{d}\to \mathbb {R}$ and $c\in \mathbb {R}$, denote by $\{f\geq c\}$ the set $\{x\in E\colon f(x) \geq c\}$. Similarly, we define the sets $\{f\leq c\}$ and $\{f=c\}$.

Denote by $S^{d-1}$ the $d-1$ dimensional unit sphere $\{x\in\mathbb{R}^{d} \colon \vert x\vert = 1\}$; for $\varepsilon> 0$ and $x\in\mathbb{R}^{d}$, $B^{d}(x, \varepsilon):=\{y\in\mathbb{R}^{d}\colon \vert x-y\vert \leq\varepsilon\}$; $B^{d}(\varepsilon):= B^{d}(\theta,\varepsilon)$ and $B^{d}:=B^{d}(1)$.

By $\mathbb{P}^{d-1}$ we denote the $d-1$ dimensional real projective space, i.e., the set of all lines in $\mathbb{R}^{d}$ that contain *θ*. The distance between two lines $r_{1},r_{2}\in\mathbb{P}^{d-1}$ is by definition equal to the angle between $r_{1}$ and $r_{2}$. The measure of a set $A\subset \mathbb{P}^{d-1}$ is by definition equal to the spherical measure of the set $\bigcup_{l\in A}l\cap S^{d-1}$; so that the measure of $\mathbb{P}^{d-1}$ is equal to the measure of $S^{d-1}$.

For each $r\in\mathbb{P}^{d-1}$, by $\Pi^{d-1}(r)$ we denote the hyperplane that contains *θ* and is orthogonal to the line *r*; $\Pi^{d-1}(r)$ is considered as a $d-1$-dimensional space with $d-1$-dimensional Lebesgue measure and Euclidean metric. For each $\beta\in\Pi^{d-1}(r)$, by $l(r,\beta)$ we denote the line that contains *β* and is parallel to *r*.

It is assumed that product topology is induced on the Cartesian product of a finite number of topological spaces and product measure is induced on the Cartesian product of a finite number of measure spaces. By $\mu^{k}$, $k\in \mathbb {N}$, we denote the *k*-dimensional Lebesgue measure in $\mathbb {R}^{k}$; by *μ* we denote the spherical measure on the unit sphere $S^{d-1}$ and the measure on the projective space $\mathbb{P}^{d-1}$.

## Variation of a set

### Definition

#### Definition 1

Denote by $N(F)$ the number of connected components of the set $F\subset\mathbb{R}^{d}$; 0 for an empty set, and +∞ if the set of connected components is infinite.

Variation of a set *F* determined by $r\in\mathbb{P}^{d-1}$ is defined by the following formula.

#### Definition 2

For a compact set $F\subset\mathbb{R}^{d}$ and a line $r\in\mathbb {P}^{d-1}$, set

$$v(F, r):= \mathop {\operatorname {ess\,sup}}_{\beta\in\Pi^{d-1}(r)}N \bigl( F\cap l(r, \beta) \bigr). $$

#### Definition 3

For a compact set $F\subset\mathbb{R}^{d}$ and a number $p\in[1,\infty ]$, set

$$v_{p}(F):= \textstyle\begin{cases} ( \frac{1}{\mu\mathbb{P}^{d-1}}\int _{\mathbb {P}^{d-1}} v^{p}(F,r)\,dr) ^{ \frac{1}{p}}, & p\in[1,\infty), \\ \mathop {\operatorname {ess\,sup}}_{r\in\mathbb{P}^{d-1}} v(F,r), & p=\infty. \end{cases} $$

#### Remark 1

If $d=1$, then for all $p\in[1,\infty]$ we set $v_{p}(F) = N(F)$.

#### Definition 4

Let a compact set $F\subset\mathbb{R}^{d}$, $\varepsilon\geq0$ and $p\in[1,\infty]$ be given. Set

$$V_{p}^{\varepsilon}(F):= \sup\sum_{k=1}^{n} v_{p}(F_{k}), $$

where the supremum is taken over all partitions $F=\bigcup_{k=1} ^{n}F_{k}$ of the set *F* into a finite number of compact pairwise *ε*-disjoint subsets $F_{k}$.

#### Remark 2

We write $V_{p}(F)$ instead of $V_{p}^{0}(F)$. In this case the supremum in the definition is taken over all partitions $F=\bigcup_{k=1} ^{n}F_{k}$ of the set *F* into a finite number of compact disjoint subsets $F_{k}$.

### Correctness of the definitions

The proofs of measurability of the functions that stay under integral signs (Lemmas 1-5) use ideas from [5] (Chapters 2-5) and [6] (Chapter 2).

Throughout this subsection, we identify each point $(r,x)$ of the space $\mathbb{P}^{d-1}\times \mathbb {R}^{d-1} $ ($r\in\mathbb{P}^{d-1}$, $x\in \mathbb {R}^{d-1}$) with the line $l(r,\beta)$, where $\beta\in\Pi^{d-1}(r)$ is a point with coordinates *x* with respect to some orthonormal basis of $\Pi^{d-1}(r)$ (the basis of $\Pi^{d-1}(r)$ is assumed to continuously change as $r\in\mathbb{P}^{d-1}$ changes).

We need the following lemma.

#### Lemma 1

*For every Borel set* $B\subset\mathbb{R}^{d}$, *the set* $\psi(B)$ *of all* $(r,x)\in\mathbb{P}^{d-1}\times \mathbb {R}^{d-1}$ *such that* $B\cap l(r,\beta)\neq \emptyset$ *is measurable* $(\mathbb{P}^{d-1}\times \mathbb {R}^{d-1})$. *The set* $\varphi(B):= \{t\in \mathbb {R}\colon B\cap\mathbb{R}^{d}_{t}\neq\emptyset \}$ *is measurable*.

Denote by $\psi_{1}$ one of continuous maps from $[0,1]$ onto $S^{d-1}$; such a map exists due to the Hahn-Mazurkiewicz theorem; see, for example, Theorem 6.8 in [7]. Define a function $\psi_{2}\colon[0,1]\times\mathbb{R}^{d}\to\mathbb {P}^{d-1}\times \mathbb {R}^{d-1}$ using the following rule. For each $(t,y)\in[0,1]\times\mathbb {R}^{d}$, let $\psi_{2}(t,y)$ be the line $l(r,\beta)$, where $r\in\mathbb {P}^{d-1}$ is the line that contains $\psi_{1}(t)$, and *β* is such that the line $l(r,\beta)$ contains the point *y*. It is easy to see that the function $\psi_{2}$ is continuous. Then $\psi(B)=\psi_{2}(\tilde{B})$, where $\tilde{B}:=[0,1]\times B$. This means that $\psi(B)$ is a continuous image of a Borel set $\tilde{B}\subset \mathbb {R}^{d+1}$ and hence is measurable (see, for example, Theorem 94 in [8]).

The set $\varphi(B)$ is a projection of the Borel set *B* to the *t*-axis of $\mathbb{R}^{d}$, hence is measurable.

#### Definition 5

For a compact set $F\subset\mathbb{R}^{d}$ and $\varepsilon>0$, denote by $N_{\varepsilon}(F)$ the number of *ε*-components of the set *F*; 0 for an empty set.

#### Lemma 2

*Let a compact set* $F\subset\mathbb{R}^{d}$ *be given*. *Then*, *for arbitrary* $\varepsilon>0$, $N_{\varepsilon}(F)$ *is finite and* $\lim_{\varepsilon\to0}N_{\varepsilon}(F)=N(F)$.

With each *ε*-component *W* of the set *F*, associate a ball with center in an arbitrary point $w\in W$ and radius $\frac {\varepsilon }{2}$. Balls that correspond to different *ε*-components of *F* are pairwise disjoint; hence there can be only a finite number of such balls due to the boundedness of *F*.

If *F* has a finite number of connected components $F_{1},\dots, F _{n}$, then $\varepsilon_{0}:=\min_{i\neq j}\rho(F_{i},F_{j})>0$ due to the compactness of components of a compact set. Then $N_{\varepsilon}(F) = n=N(F)$ for all $\varepsilon< \varepsilon_{0}$.

If $N(F)=\infty$, then for arbitrary $n\in \mathbb {N}$ we can choose compact disjoint sets $F_{1},\dots, F_{n}$ such that $F=\bigcup_{k=1} ^{n}F_{k}$. Then $N_{\varepsilon}(F)\geq n$ for all $\varepsilon< \min_{i\neq j}\rho(F_{i},F_{j})$.

For a compact set $F\subset\mathbb{R}^{d}$ and $\varepsilon>0$, define functions

1 $$ v_{F}\colon\mathbb{P}^{d-1}\times \mathbb {R}^{d-1}\to \mathbb {R},\qquad v_{F}(r,x) = N \bigl( F \cap l(r,\beta) \bigr), $$

and

$$\begin{aligned}& v_{F}^{\varepsilon}\colon\mathbb{P}^{d-1}\times \mathbb {R}^{d-1}\to \mathbb {R},\qquad v^{\varepsilon }_{F}(r,x) = N_{\varepsilon} \bigl( F\cap l(r,\beta) \bigr), \\& N_{F}^{\varepsilon}\colon \mathbb {R}\to \mathbb {R},\qquad N_{F}^{\varepsilon}(t)=N _{\varepsilon} \bigl( F\cap\mathbb{R}^{d}_{t} \bigr). \end{aligned}$$

The following lemma holds.

#### Lemma 3

*Let a compact set* $F\subset\mathbb{R}^{d}$ *and* $\varepsilon>0$ *be given*. *The function* $v_{F}^{\varepsilon}$ *is measurable* $(\mathbb{P}^{d-1}\times \mathbb {R}^{d-1})$. *The function* $N_{F}^{\varepsilon}$ *is measurable*.

Consider the (countable) set Ω of all closed balls in $\mathbb{R}^{d}$ with rational centers and radii. Let Ω̃ be the set of all finite unions of the balls from Ω. For each $n\in \mathbb {N}$, define $\Omega_{n}$ to be the family of all sets of the form $\bigcup_{s=1}^{m} B_{i_{s}}$, where $m\geq n$, and $\{B_{i _{s}}\}_{s=1}^{m}$ is a collection of pairwise *ε*-disjoint sets from Ω̃. Then the set $\Omega_{n}$ is countable for each $n\in \mathbb {N}$.

Note that the functions $v_{F}^{\varepsilon}$ and $N_{F}^{\varepsilon }$ take only non-negative integer values. The sets $\{ v_{F}^{\varepsilon }\geq0\} = \mathbb{P}^{d-1}\times \mathbb {R}^{d-1}$ and $\{ N_{F}^{\varepsilon}\geq0 \} = \mathbb {R}$ are measurable. Suppose now *n* is a natural number. Below we prove that

2 $$ \bigl\{ v_{F}^{\varepsilon}\geq n\bigr\} = \bigcup _{\mathcal{B} = \bigcup _{s=1}^{m} B_{i_{s}}\in\Omega _{n}} \Biggl( \bigcap_{s=1}^{m} \psi(B_{i_{s}}\cap F) \Big\backslash \bigcup_{s=1}^{m} \psi(\partial B_{i_{s}}\cap F) \Biggr) $$

and

$$\bigl\{ N_{F}^{\varepsilon}\geq n\bigr\} = \bigcup _{\mathcal{B} = \bigcup _{s=1}^{m} B_{i_{s}}\in\Omega _{n}} \Biggl( \bigcap_{s=1}^{m} \varphi(B_{i_{s}}\cap F) \Big\backslash \bigcup_{s=1}^{m} \varphi(\partial B_{i_{s}}\cap F) \Biggr), $$

where the functions *ψ* and *φ* are defined in Lemma 1. We prove equality (2); the other one can be proved using similar arguments.

If $(r,x)$ belongs to the right-hand side of (2), then there exists a set $\mathcal{B} = \bigcup_{s=1}^{m} B_{i_{s}} \in\Omega_{n}$ such that $l(r,\beta)\cap B_{i_{s}}\cap F$ is a non-empty set strictly inside $B_{i_{s}}$, $s=1,\dots, m$. Since the sets that constitute $\mathcal{B}$ are *ε*-disjoint, by the definition of $\Omega_{n}$, we obtain that $v_{F}^{\varepsilon}(r,x) \geq m\geq n$ and hence $(r,x)\in\{v_{F}^{\varepsilon}\geq n\}$.

Let $(r,x)\in\{v_{F}^{\varepsilon}\geq n\}$ and $F_{1},\dots, F _{m}$, $m\geq n$ be all *ε*-components of the set $l(r,\beta)\cap F$. Since $\min_{i\neq j}\rho(F_{i},F_{j})> \varepsilon$, there exists $\delta>0$ such that $\min_{i \neq j}\rho(F_{i},F_{j})>\varepsilon+ 3\delta$. Consider a finite cover *C* of the compact set $l(r,\beta)\cap F$ by open balls with rational centers and radii such that each ball has diameter less than *δ* and contains some point from $l(r,\beta)\cap F$. Denote by $B_{k}$ the union of closures of all balls from the cover *C* that intersect $F_{k}$, $k=1,\dots, m$. Then $\bigcup_{k=1}^{m}B _{k}$ belongs to $\Omega_{n}$ by construction, and hence $(r,x)$ belongs to the right-hand side of (2).

Since *F* is a closed set and $\Omega_{n}$ is a countable set, Lemma 1 implies that the sets $\{v_{F}^{\varepsilon}\geq n\}$ and $\{N_{F}^{\varepsilon}\geq n\}$ are measurable; hence the functions $v_{F}^{\varepsilon}$ and $N_{F}^{\varepsilon}$ are measurable.

#### Lemma 4

*The function* $v_{F}$ *defined by* (1) *is measurable* $(\mathbb{P}^{d-1}\times \mathbb {R}^{d-1})$ *for each compact* $F\subset \mathbb{R}^{d}$.

For each fixed $(r,x)\in\mathbb{P}^{d-1}\times \mathbb {R}^{d-1}$, one has $\lim_{\varepsilon\to0}v_{F}^{\varepsilon}(r,x)=v_{F}(r,x)$ due to Lemma 2. Hence the measurability of $v_{F}$ is a consequence of the measurability of $v_{F}^{\varepsilon}$, $\varepsilon>0$, stated in Lemma 3.

Tonelli’s theorem and Lemma 4 imply that the function $v_{p}(F)$ is well defined for every $1\leq p\leq\infty$ and compact $F\subset\mathbb{R}^{d}$; hence the functions $V_{p}^{\varepsilon}(F)$ are also well defined for all $\varepsilon\geq0$.

### Some properties of the sets variation

The following property is a direct consequence of the definitions.

#### Property 1

Let $p\in[1,\infty]$, $\varepsilon\geq0$ and $F\subset\mathbb {R}^{d}$ be a compact set. Then $v_{p}(F)\leq V_{p}^{\varepsilon}(F)$. If *F* is *ε*-connected, then $v_{p}(F)= V_{p}^{\varepsilon}(F)$.

#### Property 2

Let $p\in[1,\infty]$ and $F\subset\mathbb{R}^{d}$ be a compact set. Then

3 $$ V_{p}(F) = \lim_{\varepsilon\to0} V_{p}^{\varepsilon}(F). $$

From the definition it follows that $V_{p}^{\varepsilon_{1}}(F)\leq V _{p}^{\varepsilon_{2}}(F)$ whenever $\varepsilon_{1}>\varepsilon_{2}$. This implies that $\lim_{\varepsilon\to0} V_{p}^{\varepsilon }(F)$ exists and does not exceed $V_{p}(F)$.

Assume that $V_{p}(F)<\infty$. Then, for arbitrary $\delta>0$, there exists a partition $F=\bigcup_{k=1}^{n(\delta)} F_{k}$ of the set *F* into pairwise disjoint compact sets $F_{k}$ such that $V_{p}(F)\leq\sum_{k=1}^{n(\delta)}v_{p}(F_{k})+\delta$. Set $\varepsilon_{0} :=\min_{i\neq j}\rho(F_{i},F_{j})$. Then $\varepsilon_{0}>0$ and for all $\varepsilon<\varepsilon_{0}$ $V_{p}^{\varepsilon}(F)\geq V_{p}(F)-\delta$. This implies (3).

The case when $V_{p}^{\varepsilon}(F)=\infty$ can be considered in a similar way.

#### Property 3

Let $n\in \mathbb {N}$ and pairwise disjoint compact sets $F_{1},\dots,F _{n}\subset\mathbb{R}^{d}$ be given. Then, for all $p\in[1,\infty]$, $v_{p} ( \bigcup_{k=1}^{n}F_{k} ) \leq\sum_{k=1} ^{n}v_{p}(F_{k})$.

It is sufficient to prove the property in the case $n=2$. Set $F:=F_{1}\cup F_{2}$.

Since $F_{1}$ and $F_{2}$ are compact disjoint sets, we have $\rho(F_{1},F_{2})>0$, and hence, for arbitrary $r\in\mathbb {P}^{d-1}$ and $\beta\in\Pi^{d-1}(r)$, one has $N(F\cap l(r,\beta)) = N(F_{1} \cap l(r,\beta)) + N(F_{2}\cap l(r,\beta))$. This implies that $v(F,r)\leq v(F_{1},r) + v(F_{2},r)$ for all $r\in\mathbb{P}^{d-1}$, and hence $v_{p}(F)\leq v_{p}(F_{1})+v_{p}(F_{2})$ for all $p\in[1,\infty]$.

#### Property 4

Assume $n\in \mathbb {N}$, $\varepsilon\geq0$ and pairwise *ε*-disjoint compact sets $F_{1},\dots,F_{n}\subset\mathbb{R}^{d}$ are given. Then, for all $p\in[1,\infty]$,

$$V_{p}^{\varepsilon} \Biggl( \bigcup_{k=1}^{n}F_{k} \Biggr) = \sum_{k=1}^{n}V_{p}^{\varepsilon}(F_{k}). $$

It is sufficient to prove the property in the case $n=2$. Set $F:=F_{1}\cup F_{2}$.

Let $\{T_{k}\}_{k=1}^{m}$, $m\in \mathbb {N}$, be a partition of the set *F* into compact pairwise *ε*-disjoint subsets. Then, by Property 3,

$$\begin{aligned} \sum_{k=1}^{m}v_{p}(T_{k}) =& \sum_{k=1}^{m}v_{p} \bigl((T_{k} \cap F_{1})\cup(T_{k}\cap F_{2})\bigr) \\ \leq&\sum_{k=1}^{m}v_{p}(T_{k} \cap F_{1})+\sum_{k=1} ^{m}v_{p}(T_{k} \cap F_{2}) \\ \leq& V_{p}^{\varepsilon}(F_{1})+V_{p}^{ \varepsilon}(F_{2}) \end{aligned}$$

since $\{T_{k}\cap F_{1}\}_{k=1}^{m}$ and $\{T_{k}\cap F_{2}\}_{k=1} ^{m}$ are partitions of the sets $F_{1}$ and $F_{2}$ into pairwise *ε*-disjoint compact subsets. This implies that

$$V_{p}^{\varepsilon}(F)\leq V_{p}^{\varepsilon }(F_{1})+V_{p}^{\varepsilon }(F_{2}). $$

On the other hand, for arbitrary partitions of the sets $F_{1}$ and $F_{2}$ into compact *ε*-disjoint sets $\{T_{k}^{1}\}_{k=1} ^{s}$ and $\{T_{k}^{2}\}_{k=1}^{m}$ respectively, $s,m\in \mathbb {N}$,

$$\bigl\{ T_{k}^{1}\bigr\} _{k=1}^{s}\cup \bigl\{ T_{k}^{2}\bigr\} _{k=1}^{m} $$

is a partition of the set *F* into compact *ε*-disjoint sets, and hence

$$V_{p}^{\varepsilon}(F)\geq V_{p}^{\varepsilon }(F_{1})+V_{p}^{\varepsilon }(F_{2}). $$

#### Property 5

If $\varepsilon\geq0$ and $F\subset\mathbb{R}^{d}$ is a compact set that has exactly $n\in \mathbb {N}$ *ε*-connected components $F_{1},\dots, F_{n}$, then, for all $p\in[1,\infty]$, $V_{p}^{\varepsilon}(F) = \sum_{k=1}^{n} v_{p}(F_{k})$.

Note that each *ε*-connected component of a compact set is compact. Hence, by Properties 4 and 1, $V_{p}^{\varepsilon}(F) = \sum_{k=1}^{n} V_{p}^{\varepsilon}(F _{k})=\sum_{k=1}^{n} v_{p}(F_{k})$.

#### Property 6

If $F\subset\mathbb{R}^{d}$ is a compact set, $\alpha\neq0$ and $\alpha F:= \{\alpha x\colon x\in F\}$, then for arbitrary $p\in[1,\infty]$ $v_{p}(F) = v_{p}(\alpha F)$ and $V_{p}(F) = V_{p}(\alpha F)$.

The property follows from the observation that for arbitrary $r\in\mathbb{P}^{d-1}$ and $\beta\in\Pi^{d-1}(r)$ one has $N(F\cap l(r,\beta))= N(\alpha F\cap l(r,\alpha\beta))$ and hence $v(\alpha F,r)= v(F,r)$.

## Variation of a function

### Definition

#### Definition 6

Let a set $E\subset\mathbb{R}^{d}$ and a function $f\colon E\to \mathbb {R}$ be given. For $t\in \mathbb {R}$, the set

$$L(f;t):=\bigl\{ x\in E\colon f(x)=t\bigr\} $$

is called a level set of the function *f*.

The variation of a continuous function is given by the following definition.

#### Definition 7

Let $E\subset\mathbb{R}^{d}$, $f\colon E\to \mathbb {R}$ be a continuous on a compact subset $F\subset E$ function, and $p\in[1,\infty]$. Set

$$v_{p}(f;F):= \int _{-\infty}^{\infty}v_{p}\bigl(F\cap L(f;t) \bigr)\,dt. $$

If *F* is locally connected, then set

$$V_{p}(f;F):= \int _{-\infty}^{\infty}V_{p}\bigl(F\cap L(f;t) \bigr)\,dt. $$

If $F=E$, then we write $v_{p}(f)$ and $V_{p}(f)$ instead of $v_{p}(f;E)$ and $V_{p}(f;E)$, respectively.

### Correctness of the definitions

We need to prove that the functions under the integral signs are measurable.

#### Lemma 5

*Let* $E\subset\mathbb{R}^{d}$, $f\colon E\to \mathbb {R}$ *be a continuous on a compact subset* $F\subset E$ *function*, *and* $p\in[1,\infty]$. *Then the function* $v_{p}(F\cap L(f;\cdot))$ *is measurable*.

Without loss of generality, we may assume that $\mathbb{R}^{d}\supset F=E$, and we need to prove that the function $v_{p}(L(f;\cdot))$ is measurable. Consider the graph

4 $$ \Gamma(f):=\bigl\{ (x,t)\in \mathbb {R}^{d+1}\colon x\in E, f(x)=t \bigr\} $$

of the function *f* and two functions $v_{ \Gamma(f)}\colon \mathbb{P}^{d}\times\mathbb{R}^{d}\to \mathbb {R}$ and $v_{f}\colon \mathbb {R}\times\mathbb{P}^{d-1} \times \mathbb {R}^{d-1}\to \mathbb {R}$ defined by the formula $v_{f}(t,r,x) = v _{L(f;t)}(r,x)$ (see (1) for the definition of the function $v_{F}$).

Since the set *E* is compact and the function *f* is continuous on *E*, the set $\Gamma(f)\subset \mathbb {R}^{d+1}$ is compact. This, by Lemma 4, implies that the function $v_{ \Gamma (f)}$ is measurable $(\mathbb{P}^{d}\times\mathbb{R}^{d})$.

Recall that $\mathbb {R}^{d+1}_{0}= \{ (x,0)\in \mathbb {R}^{d+1}\colon x\in\mathbb {R}^{d} \} $. The function

$$\phi\colon \mathbb {R}\times\mathbb{P}^{d-1}\times \mathbb {R}^{d-1}\to\mathbb {P}^{d}\times \mathbb{R}^{d}\cap \bigl\{ (R,y)\in \mathbb{P}^{d}\times\mathbb {R}^{d}\colon R\subset \mathbb {R}^{d+1}_{0} \bigr\} $$

that maps a point $(t,r,x)$ to the point $(R,y)$ with $R=\{(z,0), z \in r\}$ and $y=(x,t)$ is continuous and has continuous inverse. Moreover, for arbitrary $(t,r,x)\in \mathbb {R}\times\mathbb{P}^{d-1}\times \mathbb {R}^{d-1}$, we obtain $v_{f}(t,r,x) = v_{ \Gamma(f)}(\phi(t,r,x))$. Hence, for arbitrary $c\in \mathbb {R}$, the function *ϕ* maps the set

$$\bigl\{ (t,r,x)\in \mathbb {R}\times\mathbb{P}^{d-1}\times \mathbb {R}^{d-1}\colon v_{f}(t,r,x) \geq c \bigr\} $$

and the set

$$\bigl\{ (R,y)\in\mathbb{P}^{d}\times\mathbb{R}^{d}\colon v_{ \Gamma (f)}(R,y) \geq c \bigr\} \cap \bigl\{ (R,y)\in\mathbb{P}^{d} \times\mathbb {R}^{d}\colon R \subset \mathbb {R}^{d+1}_{0} \bigr\} . $$

The latter is an intersection of a measurable (due to measurability of $v_{ \Gamma(f)}$) set and a closed set; hence the former is also a measurable set. This means that the function $v_{f}$ is measurable $(\mathbb {R}\times\mathbb{P}^{d-1}\times \mathbb {R}^{d-1})$, and hence the statement of the lemma is true due to Tonelli’s theorem.

The following result is well known (see, for example, [9], Lemma 4, or [6], Lemma 3 in Chapter 5).

#### Lemma 6

*For an arbitrary function* $f\colon E\to \mathbb {R}$, *denote by* $T_{\mathrm{extr}}$ *the set of* $t\in \mathbb {R}$ *such that* $L(f;t)$ *contains an extremum point*. *Then* $T_{\mathrm{extr}}$ *is at most countable*.

#### Lemma 7

*Let* $E\subset\mathbb{R}^{d}$, $f\colon E\to \mathbb {R}$ *be a continuous on a compact locally connected subset* $F\subset E$ *function*, *and* $p\in[1,\infty]$. *Then the function* $V_{p}(F\cap L(f;\cdot))$ *is measurable*.

Without loss of generalization, we may assume that $F=E$. Taking into account Property 2, it is sufficient to prove that each of the functions $V_{p}^{\nu}(L(f;\cdot))$ is measurable, $\nu>0$.

Let $\nu>0$ be fixed. The function $N_{f}(t):=N_{\nu}(L(f;t))=N_{ \Gamma(f)}^{\nu}(t)$ is measurable due to Lemma 3 ($\Gamma(f)$ is defined by (4)). Due to Lemma 6, for each $k= 0,1, \dots$, the set $T_{k}:=\{N_{f} =k\}\setminus T_{\mathrm{extr}}$ is measurable; moreover, obviously these sets are pairwise disjoint.

Let $c\in \mathbb {R}$ be given. Then

$$\bigl\{ V_{p}^{\nu}\bigl(L(f;\cdot)\bigr)\leq c\bigr\} \setminus T_{\mathrm{extr}}=\bigcup_{k\in \mathbb {N}}\bigl\{ t\in T_{k}\colon V_{p}^{\nu}\bigl(L(f;t)\bigr)\leq c\bigr\} $$

and it is sufficient to prove that for each $k\in \mathbb {N}$ the set $\{t\in T_{k}\colon V_{p}^{\nu}(L(f;t))\leq c\}$ is measurable.

Let some $k\in \mathbb {N}$ and $t^{*}\in T_{k}$ be fixed. The set $L(f;t^{*})$ contains exactly *k* *ν*-connected components $F_{1},\dots, F_{k}$. Each of the components is a compact set, hence there exists $\varepsilon>0$ such that the sets $U_{s}(\varepsilon)= \{x\colon\rho(x,F_{s})<\varepsilon\}$, $s=1,\dots, k$, are pairwise *ν*-disjoint. Set $U(\varepsilon):=\bigcup_{s=1}^{k} U_{s}( \varepsilon)$.

There exists $\delta>0$ such that $L(f;t)\subset U(\varepsilon)$ for all $t\in(t^{*}-\delta, t^{*}+\delta)$. Really, assume the contrary, suppose that there exists a sequence $a_{n}$, $a_{n}\to0$ as $n\to\infty$, such that each of the level sets $L(f;t^{*}+a_{n})$ contains a point $x_{n}\notin U(\varepsilon)$. Switching to a subsequence, if needed, we may assume that the sequence $x_{n}$ converges to some $x\in\mathbb{R}^{d}$. Since $U(\varepsilon)$ is an open set, $x\notin U(\varepsilon)$. However, this is impossible since *f* is continuous, and hence $f(x) = t^{*}$.

For each $s=1,\dots, k$, consider an arbitrary point $x_{s}\in F_{s}$. Since the level set $L(f,t^{*})$ does not contain extremums, *F* is locally connected and *f* is continuous; for small enough $\varepsilon _{s}>0$, the set $f ( B^{d}(x_{s},\varepsilon_{s})) $ contains a neighborhood $(t^{*}-\delta_{s},t^{*}+\delta_{s})$ of $t^{*}$ ($\delta_{s}>0$). Hence, for arbitrary $t\in(t^{*}-\delta_{s},t^{*}+ \delta_{s})$, the level set $L(f;t)$ contains at least one *ν*-component inside $U_{s}(\varepsilon)$. This means that there exists $\delta=\delta(t^{*})>0$ such that each of the level sets $L(f,t)$, $t\in(t^{*}-\delta, t^{*}+\delta)$ contains at least one *ν*-component inside $U_{s}(\varepsilon)$, $s=1,\dots, k$. Hence, for all $t\in T_{k}\cap(t^{*}-\delta, t^{*}+\delta)$, the level set $L(f;t)$ contains exactly one *ν*-component inside $U_{s}(\varepsilon)$, $s=1,\dots, k$. This implies that for all $t\in T_{k}\cap(t^{*}- \delta, t^{*}+\delta)$

5 $$ V_{p}^{\nu}\bigl(L(f;t)\bigr)=\sum _{s=1}^{k}v_{p} \bigl( L(f;t)\cap \overline{U _{s}(\varepsilon)} \bigr) $$

due to Property 5.

For each $t^{*}\in T_{k}$, set $W(t^{*}):=(t^{*}-\delta(t^{*}),t^{*}+ \delta(t^{*}))$; the sets $W(t^{*})$, $t^{*}\in T_{k}$, constitute an open cover of the set $T_{k}$. Since $\mathbb {R}$ is a Lindelöf space, we can find a countable subcover $W_{1},W_{2},\dots$ of $T_{k}$. Set $\tilde{W}_{1}:=W_{1}\cap T_{k}$, $\tilde{W}_{m}:= ( W_{m}\setminus \bigcup_{s=1}^{m-1}W_{s} ) \cap T_{k}$, $m=2,3,\dots$. We obtain a countable partition of the set $T_{k}$ into pairwise disjoint measurable subsets $\tilde{W}_{m}$, $m\in \mathbb {N}$, such that on each of the sets $\tilde{W}_{m}$ we have representation (5) of the function $V_{p}^{\nu}(L(f;\cdot))$ (the sets $\overline{U _{s}(\varepsilon)}$, $s=1,\dots, k$, might be different for different $m\in \mathbb {N}$). Due to Lemma 5, this implies the measurability of the set

$$\bigl\{ t\in T_{k}\colon V_{p}^{\nu}\bigl(L(f;t) \bigr)\leq c\bigr\} = \bigcup_{m\in \mathbb {N}} \Biggl( \tilde{W}_{m}\cap \Biggl\{ \sum_{s=1} ^{k}v_{p} \bigl( L(f;t)\cap\overline{U_{s}( \varepsilon)} \bigr) \leq c \Biggr\} \Biggr) $$

and hence the lemma is proved.

### Some properties of the function variation

Below we list some properties of the function variation. Everywhere $p\in[1,\infty]$ and a compact set $F\subset\mathbb{R}^{d}$ are fixed, *f* is continuous on *F* function. For properties of $V_{p}(f)$, the set *F* is further assumed to be locally connected.

#### Property 7

$v_{p}(f)$ and $V_{p}(f)$ are non-negative. If *f* is constant, then $v_{p}(f)=V_{p}(f)=0$.

The fact that variations are non-negative follows from the definition. If *f* is constant, then it has exactly one non-empty level set.

#### Property 8

If $\alpha, \beta\in \mathbb {R}$, then $v_{p}(\alpha\cdot f+\beta)=\vert \alpha \vert v_{p}(f)$ and $V_{p}(\alpha\cdot f+\beta)=\vert \alpha \vert V_{p}(f)$.

Note that $L(\alpha\cdot f + \beta;t) = L(f; \alpha^{-1}\cdot(t- \beta))$ for all $\alpha\neq0$ and $\beta, t\in \mathbb {R}$. Making substitution $s=\alpha^{-1}\cdot(t-\beta)$ in the integrals from Definition 7, we obtain the required equalities. In the case $\alpha=0$, the property follows from Property 7.

#### Property 9

For arbitrary $\alpha\neq0$, $v_{p}(f; F)= v_{p} ( f(\alpha \cdot); \alpha^{-1}F ) $ and

$$V_{p}(f; F) = V_{p}\bigl(f(\alpha \cdot); \alpha^{-1}F\bigr). $$

The property follows from Property 6.

#### Property 10

Let $t\in \mathbb {R}$ be such that $F\setminus L(f;t)$ has exactly $n\geq2$ connected components $F_{1},F_{2},\dots, F_{n}$. Assume that for all $k=1,\dots, n$, $\overline{F_{k}}\setminus F_{k}\subset L(f;t)$. Then $v_{p}(f;F) \leq\sum_{k=1}^{n}v_{p}(f;\overline{F_{k}})$ and $V_{p}(f;F) = \sum_{k=1}^{n}V_{p}(f;\overline{F_{k}})$.

Consider arbitrary $s\in \mathbb {R}$, $s\neq t$. For $k=1,\dots, n$, set $W_{k}:=\overline{F_{k}}\cap L(f;s)$. Then the set $W_{k}$ is closed, $k=1,\dots, n$, and $W_{k}\subset F_{k}$ due to conditions of the property and the fact that different level sets of any function are disjoint. This means that $W_{k}$ are compact pairwise disjoint sets. From Properties 3 and 4 it follows that $V_{p}(L(f;s)) = \sum_{k=1}^{n}V_{p}(\overline{F_{k}}\cap L(f;s))$ and $v_{p}(L(f;s)) \leq\sum_{k=1}^{n}v_{p}(\overline{F_{k}}\cap L(f;s))$. The statement of the property now follows from Definition 7.

#### Property 11

If $d=1$ and $f\colon[0,1]\to \mathbb {R}$ is a continuous function, then for all $p\in[1,\infty]$

$$v_{p}\bigl(f;[0,1]\bigr) = V_{p}\bigl(f;[0,1]\bigr) = \bigvee_{0}^{1}f. $$

#### Remark 3

$\bigvee_{0}^{1}f$ is the classical variation of a univariate function *f* on $[0,1]$. We allow $\bigvee_{0}^{1}f$ to be +∞ in the case when *f* is not a function of bounded variation.

The Banach indicatrix theorem [10] states that $\bigvee_{0}^{1}f$ is equal to the integral over $t\in \mathbb {R}$ of number of points in $L(f;t)$. In the definition of $v_{p}(f;[0,1])$, the number of components of $L(f;t)$ is integrated over $t\in \mathbb {R}$. Each component of a compact set in $\mathbb {R}$ is a point or a segment. The family of level sets $L(f;t)$ that contain a segment as a connected component is at most countable because each of such level sets contains extremum (see Lemma 6). Hence $v_{p}(f;[0,1]) = \bigvee_{0}^{1}f$. It is easy to see that $v_{p}(f;[0,1]) = V_{p}(f;[0,1])$.

#### Property 12

Let $\varphi\colon[0,1]\to \mathbb {R}$ be a continuous function and $d\in \mathbb {N}$. Let $f_{\varphi}\colon B^{d}\to \mathbb {R}$, $f_{\varphi}(x) = \varphi(\vert x\vert )$. Then, for all $p\in[1,\infty]$,

$$v_{p}\bigl(f_{\varphi};B^{d}\bigr) = V_{p} \bigl(f_{\varphi};B^{d}\bigr) = 2\cdot\bigvee _{0}^{1}\varphi. $$

In the case $d=1$, the property follows from Property 11, so we can assume that $d\geq2$. Let arbitrary $t\neq\varphi(0)$ be fixed. For arbitrary $r\in\mathbb {P}^{d-1}$ and $\beta\in\Pi^{d-1}(r)$ the number $N(L(f_{\varphi};t)\cap l(r, \beta))$ can be obtained by the following procedure: consider the line $r=l(r,\theta)$ and mark points of the set $L(f_{\varphi};t)\cap l(r, \theta)$; cut the interval $(-\vert \beta \vert ,\vert \beta \vert )$ from the line and stick the points $-\vert \beta \vert $ and $\vert \beta \vert $ together; the number of components of marked points on the obtained ‘cut’ line is equal to $N(L(f_{\varphi};t)\cap l(r,\beta))$. This shows that for arbitrary *β*

6 $$ N\bigl(L(f_{\varphi};t)\cap l(r,\beta)\bigr)\leq N \bigl(L(f_{\varphi};t)\cap l(r, \theta)\bigr). $$

From the choice of *t* it follows that

7 $$ \theta\notin L(f_{\varphi};t), $$

and hence there exists $\varepsilon>0$ such that $B(\varepsilon) \cap L(f_{\varphi};t) =\emptyset$. This implies that the set $L(f_{\varphi};t)\cap l(r,\theta)$ does not contain points *x* with $\vert x\vert <\varepsilon$ and hence for all *β* such that $\vert \beta \vert < \varepsilon$ (6) becomes equality. This implies that $v(L(f_{\varphi};t), r) = N(L(f_{\varphi};t)\cap l(r,\theta))$. From (7) it follows that $N(L(f_{\varphi};t) \cap l(r,\theta)) = 2\cdot N(L(\varphi; t))$. Equality $v_{p}(f _{\varphi};B) = 2\cdot\bigvee_{0}^{1}\varphi$ follows from Property 11 now. Equality $v_{p}(f_{\varphi};B) = V_{p}(f_{\varphi};B)$ follows from the geometry of the level sets of $f_{\varphi}$.

## Ostrowski type inequalities

### Auxiliary results

#### Lemma 8

*Let* $d\in \mathbb {N}$, $d\geq2$, $\varepsilon>0$, $x\in\mathbb{R}^{d}$, $r\in\mathbb{P}^{d-1}$ *and a measurable set* $F\subset B^{d}(x,\varepsilon)$ *be given*. *For arbitrary* $A\in(0,1)$, *there exists* $\alpha=\alpha(A)\in(0,1)$ *that does not depend on* *ε*, *x* *and* *r* *such that*

$$\mu^{d-1}\bigl\{ \beta\in\Pi^{d-1}(r)\colon F\cap l(r,\beta)\neq \emptyset \bigr\} >A\cdot\mu^{d-1}B^{d-1}(\varepsilon) $$

*whenever* $\mu^{d}F >\alpha\cdot\mu^{d}B^{d}(\varepsilon)$.

The fact that *α* does not depend on *ε* follows from the observation that

$$\frac{\mu^{d}F}{\mu^{d}B^{d}(\varepsilon)} = \frac{\mu^{d} ( \frac{1}{ \varepsilon}F ) }{\mu^{d}B^{d}} $$

and

$$\frac{\mu^{d-1}\{\beta\in\Pi^{d-1}(r)\colon F\cap l(r,\beta)\neq \emptyset\}}{\mu^{d-1}B^{d-1}(\varepsilon)} = \frac{\mu^{d-1}\{ \beta\in\Pi^{d-1}(r)\colon\frac{1}{\varepsilon}F\cap l(r,\beta) \neq\emptyset\}}{\mu^{d-1}B^{d-1}}. $$

The fact that *α* is independent of *x* and *r* is obvious. The existence of *α* follows from the equality

8 $$ \mu^{d} F = \int _{\Pi^{d-1}(r)\cap B^{d}(y, \varepsilon)}\mu ^{1}\bigl(l(r,\beta)\cap F\bigr) \mu^{d-1}(d\beta), $$

where $y\in\Pi^{d-1}(r)$ is such that the line $l(r,y)$ contains *x* and equality $\mu^{d-1}(\Pi^{d-1}(r)\cap B^{d}(y, \varepsilon)) = \mu^{d-1}B^{d-1}$.

#### Lemma 9

*Let* $p\in[1,\infty)$, $A>0$ *and* $B\in[0,A]$ *be given*. *Then*

$$\frac{1}{A} \bigl( B + 2^{p}(A-B) \bigr) \geq \biggl( 2- \frac {B}{A} \biggr) ^{p}. $$

It is sufficient to prove that the function $\varphi(x) = 2^{p} + (1-2^{p})x - (2-x)^{p}$ is non-negative on $[0,1]$. Since $\varphi(0)= \varphi(1)=0$, the function $\varphi'$ has at least one zero on $(0,1)$. The function $\varphi'(x) = p(2-x)^{p-1} + 1 - 2^{p}$ is decreasing on $[0,1]$, hence has at most one zero on $(0,1)$. This implies that $\varphi'(0)>0$ and hence the function *φ* is increasing on $[0,x^{*}]$ and is decreasing on $[x^{*},1]$, where $x^{*}$ is zero of $\varphi'$ on $(0,1)$; hence *φ* is non-negative on $[0,1]$.

### Main results

The following theorem is the main tool to prove Ostrowski type inequalities for functions and sets of bounded variation below.

#### Theorem 1

*Let* $d\in \mathbb {N}$ *and two sets* $F,W\subset B^{d}$ *be given*. *Assume that the following properties hold*:

1. *F* *is measurable and* $\theta\notin\overline{F}$;
2. *W* *is closed and* $\theta\notin W$; *and*
3. *If* $x\in F$ *and* $y\in B^{d}\setminus F$, *then* $xy\cap W\neq\emptyset $.

*Then*, *for all* $p\in[1,\infty]$,

9 $$ \mu^{d} F \leq\frac{\mu^{d} B^{d}}{2} v_{p}(W). $$

*The inequality is sharp in the sense that for arbitrary* $\varepsilon >0$ *there exist sets* *F* *and* *W* *that satisfy conditions above and such that*

$$\mu^{d} F > \biggl( \frac{\mu^{d} B^{d}}{2}-\varepsilon \biggr) v_{p}(W). $$

*If* (9) *becomes equality*, *then* $\mu^{d} F=0$.

We will prove Theorem 1 in the next subsections. Here we state two consequences of this theorem, which can be considered as Ostrowski type inequalities.

#### Theorem 2

*Let* $d\in \mathbb {N}$ *and a continuous function* $f\colon B^{d}\to \mathbb {R}$ *be given*. *Then*, *for all* $p\in[1,\infty]$,

$$\biggl\vert \frac{1}{\mu^{d} B^{d}} \int _{B^{d}}f(x)\,dx-f(\theta)\biggr\vert \leq \frac{v_{p}(f)}{2}. $$

*The inequality is sharp*. *It becomes equality only in the case when* *f* *is constant*.

Due to Property 8, we can assume that $f(\theta) = 0$, and it is sufficient to prove that

10 $$ \int _{B^{d}}f(x)\,dx\leq\frac{\mu^{d} B^{d}}{2}v_{p}(f). $$

Consider a set

$$\Gamma:=\bigl\{ (x,t)\in B^{d}\times[0,\infty)\colon f(x)\geq t\bigr\} . $$

Then

11 $$ \int _{B^{d}}f(x)\,dx\leq\mu^{d+1}\Gamma= \int _{t\geq0} \mu^{d}\bigl(\Gamma\cap \mathbb {R}^{d+1}_{t}\bigr)\,dt. $$

For each $t >0$, consider the sets $F:=\Gamma\cap \mathbb {R}^{d+1}_{t}$ and $W:=\Gamma(f)\cap \mathbb {R}^{d+1}_{t}$ (see (4) for the definition of $\Gamma(f)$). Both *F* and *W* are closed sets that do not contain *θ* since $f(\theta) = 0$. If $x\in F$ and $y\in B^{d}\setminus F$, then $f(x)\geq t$ and $f(y)< t$ and hence the segment *xy* contains a point *z* with $f(z)=t$, i.e., $xy\cap W\neq\emptyset$. This means that all the conditions of Theorem 1 are satisfied and hence

$$\mu^{d}\bigl(\Gamma\cap \mathbb {R}^{d+1}_{t}\bigr) = \mu^{d}(F)\leq\frac{\mu^{d} B ^{d}}{2} v_{p}(W) = \frac{\mu^{d} B^{d}}{2} v_{p}\bigl(L(f;t)\bigr) $$

with equality possible only in the case when $\mu^{d} F=0$. Taking into account (11), we obtain

$$\mu^{d+1}\Gamma\leq\frac{\mu^{d} B^{d}}{2} \int _{t\geq0}v _{p}\bigl(L(f;t)\bigr)\,dt \leq \frac{\mu^{d} B^{d}}{2} \int _{t\in \mathbb {R}}v _{p}\bigl(L(f;t)\bigr)\,dt = \frac{\mu^{d} B^{d}}{2} v_{p}(f) $$

and inequality (10) is proved; moreover, due to the continuity of *f*, we obtain that equality in (10) can hold only if $f\equiv0$.

For all $\varepsilon>0$, consider the function $\varphi_{\varepsilon }\colon[0,1]\to \mathbb {R}$, $\varphi_{\varepsilon}(t) = 1$ for $t\geq\varepsilon$, $\varphi_{\varepsilon}(0)=0$ and $\varphi_{\varepsilon}$ is linear on $[0,\varepsilon]$. Due to Property 12, for the radial function $f_{\varepsilon}(x)\colon B^{d}\to \mathbb {R}$, $f_{\varepsilon}(x) = \varphi_{\varepsilon}(\vert x\vert )$, and arbitrary $p\in[1,\infty]$ $v_{p}(f_{\varepsilon}) = 2$; moreover, $\int _{B^{d}}f_{ \varepsilon}(x)\,dx\to\mu^{d} B^{d}$ as $\varepsilon\to0$. This proves the sharpness of the stated inequality.

#### Theorem 3

*Let* $d\in \mathbb {N}$ *and a closed set* $F\subset B^{d}$ *be given*. *If* $\theta\notin F$, *then for all* $p\in[1,\infty]$

$$\mu^{d} F \leq\frac{\mu^{d} B^{d}}{2} v_{p}(F). $$

*The inequality is sharp*. *If equality holds*, *then* $\mu^{d} F=0$.

It is enough to apply Theorem 1 with $W=F$; all three conditions of Theorem 1 are satisfied.

For arbitrary $\varepsilon>0$, consider a set $F_{\varepsilon}:=\{x \in B^{d}\colon \vert x\vert \geq\varepsilon\}$. For all $p\in[1,\infty]$, $v_{p}(F_{\varepsilon})=2$; $\mu^{d} F_{\varepsilon}\to\mu^{d} B ^{d}$ as $\varepsilon\to0$. This proves that the stated inequality is sharp.

#### Remark 4

In all three theorems variation $v_{p}$ can be substituted by $V_{p}$ due to Property 1. The inequalities will remain sharp.

#### Remark 5

Properties 4 and 10 state that $V_{p}$ is additive. This gives motivation to call $V_{p}$ a variation, rather than $v_{p}$.

### More auxiliary results

Denote by *F̃* the set of all points $x\in F$ such that $\lim_{\delta\to+0}\frac{\mu^{d}(F\cap B^{d}(x,\delta))}{\mu ^{d} B^{d}(\delta)} =1$. Then $\tilde{F}\cap S^{d-1}=\emptyset$ and, by the Lebesgue density theorem,

12 $$ \mu^{d}\tilde{F}=\mu^{d} F. $$

#### Lemma 10

*Assume that the conditions of Theorem* 1 *hold*. *If* $r\in\mathbb{P}^{d-1}$ *is such that* $v(W,r) = 0$, *then for arbitrary* $\beta \in\Pi^{d-1}$ *either* $\tilde{F}\supset \operatorname{int}B^{d}\cap l(r, \beta)$, *or* $\tilde{F}\cap l(r,\beta)=\emptyset$.

Assume that for some $\beta\in\Pi^{d-1}(r)$ there exist $x\in \tilde{F}\cap l(r,\beta)$ and $y\in(\operatorname{int}B^{d}\cap l(r,\beta)) \setminus\tilde{F}$. From the definition of *F̃* it follows that there exist $a>0$ and a sequence $\rho_{n}\to0$ as $n\to\infty$ such that $\mu^{d} (B^{d}(y,\rho_{n})\setminus F)\geq a\cdot\mu^{d} B^{d}( \rho_{n})$ for all $n\in \mathbb {N}$. From (8) (with *F* substituted by $B^{d}(y,\rho_{n})\setminus F$) it follows that there exists $A>0$ such that

13 $$ \mu^{d-1}\Omega_{1}(\rho_{n}) > A \cdot\mu^{d-1}B^{d-1}(\rho_{n}) $$

for all $n\in \mathbb {N}$, where

$$\Omega_{1}(\rho_{n}) = \bigl\{ \beta\in\Pi^{d-1}(r) \colon\bigl(B^{d}(y,\rho _{n})\setminus F\bigr)\cap l(r, \beta)\neq\emptyset\bigr\} . $$

Since $x\in\tilde{F}$, there exists $\delta>0$ such that for all $\rho<\delta$ one has $\mu^{d} (B^{d}(x,\rho)\cap F)\geq\alpha(1-A) \cdot\mu^{d} B^{d}(\rho)$ (the number $\alpha(1-A)$ is defined in Lemma 8). Lemma 8 implies that

14 $$ \mu^{d-1}\Omega_{2}(\rho) > (1-A)\cdot \mu^{d-1}B^{d-1}(\rho) $$

for all $\rho\leq\delta$, where

$$\Omega_{2}(\rho) = \bigl\{ \beta\in\Pi^{d-1}(r)\colon B^{d}(x,\rho) \cap F\cap l(r,\beta)\neq\emptyset\bigr\} . $$

Choose *n* so big that $\rho_{n}<\delta$. Then

15 $$ \mu^{d-1}\Omega_{1}(\rho_{n}) + \mu^{d-1}\Omega_{2}(\rho_{n}) > \mu ^{d-1}B^{d-1}(\rho_{n}) $$

due to (13) and (14). Moreover, since $x,y\in l(r,\beta)$, we receive that

16 $$ \Omega_{1}(\rho_{n}), \Omega_{2}( \rho_{n})\subset\Pi^{d-1}(r)\cap B ^{d}(\beta, \rho_{n}) $$

and

17 $$ \mu^{d-1}\bigl(\Pi^{d-1}(r)\cap B^{d}( \beta,\rho_{n})\bigr) = \mu^{d-1}\bigl(B^{d-1}( \rho_{n})\bigr). $$

Set $\Omega= \Omega_{1}(\rho_{n})\cap\Omega_{2}(\rho_{n})$. Then, due to (15), (16) and (17), $\mu^{d-1}\Omega> 0$. But each line $l(r,\beta)$, $\beta\in\Omega$, contains a point from *W* due to Condition 3 of Theorem 1 and the definitions of the sets $\Omega_{1}(\rho_{n})$ and $\Omega_{2}(\rho _{n})$; this contradicts assumption $v(W,r)=0$ of the lemma.

#### Lemma 11

*Assume that the conditions of Theorem* 1 *hold*. *Let* $R\subset\mathbb{P}^{d-1}$ *be such that* $v(W,r)=0$ *for all* $r\in R$. *If* *R* *contains* *d* *lines that are not contained in any* $d-1$-*dimensional hyperplane*, *then* $\mu^{d}(F) = 0$.

Due to (12) it is enough to prove that $\tilde{F}= \emptyset$. Let $r_{1},\dots, r_{d}$ be the lines from the statement of the lemma, and let $\rho_{1},\dots, \rho_{d}$ be unit vectors parallel to these lines. Set $P:= \{ \sum_{k=1}^{d}t_{k}\rho _{k}\colon t_{k}\in(-1,1), k=1,\dots, d \} $, then *P* is an open in $\mathbb{R}^{d}$ set.

Consider arbitrary $x\in \operatorname{int}B^{d}$. Choose $\varepsilon>0$ such that $x+\varepsilon P\subset B^{d}$. Then, for all points *y* from the segment *θx*, $P_{y} := y+\varepsilon P\subset B^{d}$. $\bigcup_{y\in\theta x}P_{y}$ is an open cover of a compact set *θx*, hence it contains a finite subcover $P_{1},P_{2},\dots, P _{m}$, $m\in \mathbb {N}$. From Lemma 10 it follows that for each $s=1,\dots, m$ either $P_{s}\subset\tilde{F}$, or

18 $$ P_{s}\cap\tilde{F}=\emptyset. $$

Since $\theta\notin\tilde{F}$, we obtain that (18) holds for each $s=1,\dots, m$ and hence $x\notin\tilde{F}$.

### Proof of Theorem 1

If $v(W,r)\geq2$ for almost all $r\in\mathbb{P}^{d-1}$, then $v_{p}(W)\geq2$ and inequality (9) holds. It is strict because Condition 1 of Theorem 1 holds. If there is a set $R\subset\mathbb {P}^{d-1}$ of positive measure such that $v(W,r) = 0$ for all $r\in R$, then $\mu^{d} F = 0$ due to Lemma 11, and inequality (9) holds.

Assume that there exists $R\subset\mathbb{P}^{d-1}$, $\mu R >0$, such that $v(W,r) = 1$ for all $r\in R$ and $v(W,r)\geq2$ for almost all $r\in\mathbb{P}^{d-1}\setminus R$. Then

19 $$ v_{p}(W) \geq2 - \frac{\mu R}{\mu S^{d-1}}. $$

Really, if $p=\infty$, then $v_{\infty}(W) \geq2$ in the case $\mu R<\mu\mathbb{P}^{d-1}$ and $v_{\infty}(W) = 1$ in the case $\mu R =\mu\mathbb{P}^{d-1}= \mu S^{d-1}$. In both cases (19) holds. In the case $p\in[1,\infty)$

$$v_{p}(W) \geq \biggl( \frac{1}{\mu S^{d-1}} \bigl( \mu R + 2^{p}\cdot\bigl( \mu S^{d-1} - \mu R\bigr) \bigr) \biggr) ^{\frac{1}{p}} \geq2 - \frac{ \mu R}{\mu S^{d-1}} $$

due to Lemma 9.

Conditions 1 and 2 of the theorem imply that there exists $\varepsilon> 0$ such that $B^{d}(\varepsilon)\cap W = \emptyset$ and $B^{d}(\varepsilon) \cap F = \emptyset$. Set $\Lambda:=\bigcup_{r\in R} (r\cap B ^{d})$. Below we prove that

20 $$ \mu^{d} (\Lambda\cap F) < \frac{\mu^{d}\Lambda}{2}. $$

In order to prove (20), it is enough to show that

21 $$ \mu^{d} (\Lambda\cap\tilde{F}) < \frac{\mu^{d}\Lambda}{2} $$

due to (12). Consider arbitrary $r\in R$. Then all points of the intersection $r\cap\tilde{F}$ are from one side of $r\cap B^{d}(\varepsilon)$. This fact can be proved using arguments similar to the proof of Lemma 10. Denote by *χ* the characteristic function of the set $\Lambda\cap \tilde{F}$. Then $\chi(x) = 0$ for all $\vert x\vert <\varepsilon$ and $\chi(x) + \chi(-x)\leq1$ for all $x\in\Lambda$. This implies (21).

Finally, having (20), we can write

$$\begin{aligned} \mu^{d}F \leq&\mu^{d}(F\cap\Lambda) + \mu^{d} \bigl(B^{d}\setminus \Lambda\bigr) \\ < & \mu^{d}B^{d}- \frac{1}{2} \mu^{d}\Lambda \\ =& \mu^{d}B^{d}-\frac{1}{2}\cdot\frac{\mu^{d}B^{d}}{\mu S^{d-1}} \mu R\\ =&\frac{\mu^{d}B^{d}}{2} \biggl( 2 - \frac{ \mu R}{\mu S^{d-1}} \biggr). \end{aligned}$$

The latter together with (19) proves (9).

The same example as in Theorem 3 shows that inequality (9) is sharp.
